# Case report: Early detection of mesenteric ischemia by intravital microscopy in a patient with septic shock

**DOI:** 10.3389/fmed.2022.985977

**Published:** 2022-08-26

**Authors:** Janina Praxenthaler, Carmen Kirchner, Elke Schwier, Simon Altmann, Axel Wittmer, Dietrich Henzler, Thomas Köhler

**Affiliations:** ^1^Department of Anesthesiology, Surgical Intensive Care, Emergency and Pain Medicine, Klinikum Herford, Ruhr University Bochum, Herford, Germany; ^2^Department of Anesthesiology, Intensive Care and Pain Medicine, Kliniken Südostbayern, Klinikum Traunstein, Traunstein, Germany; ^3^Department of General and Visceral Surgery, Thoracic Surgery and Proctology, Klinikum Herford, Ruhr University Bochum, Herford, Germany; ^4^Department of Anesthesiology, Intensive Care and Pain Medicine, Knappschaftskrankenhaus Bochum, Ruhr University Bochum, Bochum, Germany; ^5^Institute of Pathology, Klinikum Herford, Herford, Germany; ^6^Department of Anesthesiology and Intensive Care Medicine, AMEOS-Klinikum Halberstadt, Halberstadt, Germany

**Keywords:** microcirculation, intravital microscopy (IVM), acute mesenteric ischemia (AMI), septic shock, intensive care

## Abstract

Gut ischemia is a frequent but underdiagnosed complication, especially in critically ill intensive care patients, and represents a special diagnostic challenge that can only be solved in an interdisciplinary manner. We report a case of a 54-year-old woman with acute mesenteric ischemia (AMI) as a cause of septic shock diagnosed by intravital microscopy (IVM) 2 days before visible necrotic changes in a multimodality approach. We show that intravital microscopy can be a serious alternative for the early diagnosis of mesenteric ischemia in the hands of the skilled. We use this case to discuss the value and clinical perspective of IVM in the intensive care setting.

## Introduction

According to current data, mortality caused by septic shock in industrialized countries in Europe and North America decreases since 2009; however, it is still high. For example, mortality for patients with septic shock in Europe remains at approximately 40% (1, 2). Sepsis is an overwhelming host response to infection (3–5) whereby the dysregulation of complex interactions between immunological and hemostatic networks at the cellular and humoral levels leads to generalized vasopathy (6–9). This microcirculatory dysfunction contributes significantly to septic multi-organ failure and may be the main determinant of clinically relevant outcomes (10–12).

Acute mesenteric ischemia (AMI) summarizes various clinical conditions whose common feature is the interruption of intestinal perfusion. Due to the unspecific clinical symptoms, the diagnosis is often difficult and frequently delayed (13). As a result, mortality is reported to be as high as 60% (14) and up to 90% (15). Therefore, early diagnosis coupled with prompt and decisive therapy is the key to successful treatment (16).

In this context, intravital microscopy (IVM) may be a useful diagnostic tool. Sidestream dark field (SDF) imaging is a non-invasive technology by which microcirculation can be visualized *in vivo* in real-time (6, 17). The visualization of microcirculation using SDF imaging has been well-established for the oral mucosa (18). In general, it can be assumed that the perfusion ratios measured sublingually are comparable to those of the bowel serosa (19). Conversely, sublingual microcirculation may be impaired in chronic mesenteric ischemia (20). SDF imaging of the rectal mucosa has mainly been investigated in animal models. Only one study used SDF imaging to describe rectal microcirculatory alterations in humans (21). Due to limitations like limited reproducibility or high inter-observer variability microcirculation measurement has not been widely used in clinical practice (11). On the other hand, the IVM performed by a skilled examiner offers a non-invasive way to assess microcirculation in real-time.

Sidestream dark field imaging is an “image formation system” and comprises of a specifically arranged LED illumination ring around a lens system optically separated from it and a light guide. The LEDs emit pulsed illumination (intravital stroboscopy) with a wavelength of 530 nm, corresponding to the isosbestic point of the absorption spectra of oxygenated or deoxygenated blood. The final image processing and visualization are done digitally on a monitor (22).

As part of a prospective clinical study on microcirculation in septic shock (German Clinical Trials Registry DRKS00017211), we performed both sublingual and rectal SDF imaging. The rectal measurement site represents the supply area of the middle and inferior rectal artery, which originate directly or *via* the pudendal artery from the internal iliac artery. Measurements were performed and documented at both sites, sublingual and rectal, right and left, at five different time points after the onset of septic shock [0 h (T_0_), 4 h (T_1_), 8 h (T_2_), 24 h (T_3_), and 48 h (T_4_)]. To ensure comparability of measurements between all time points, we sought to maintain a mean arterial pressure of 65–85 mmHg during all measurements, as this appears to be the corridor in which autoregulatory processes keep the microcirculation at a constant level (23).

Sublingual measurements were performed according to the recommendations of the Second Consensus Conference on IVM in a grid-based approach (18). For rectal imaging, the SDF imaging probe was inserted approx. 5–7 cm rectally after cleaning, positioning the patient in the lateral position. Analysis of the rectal images was performed according to an established algorithm (21). All visible villi in a field of view (0.94 mm × 0.75 mm), perfused and not perfused, were counted and the percentage of perfused villi was calculated. In addition, the microvascular flow index (MFI) was determined as well as the De Backer score (24).

We evaluated the quality of all videos using the quality score by Massey et al. (25), with the exception of the rectal videos which show the absence of flow. We deemed the score inapplicable to those clips since the absence of flow makes it difficult to determine whether a capillary may be looped or if a pressure artifact may be present. Only videos without critical errors were taken into consideration and only the best 5–6 of each side were chosen for analysis. We achieved quality scores between 0 and 3 for a single video. Our averaged quality scores for each measurement ranged from 0.5 to 2.16, which indicate an acceptable video quality. Curiously, our worst two scores were achieved sublingually at the onset of the first septic period (score 2.16) and rectally prior to mesenteric ischemia (score 2.0), which are likely due to the edema and circulatory decline associated with these.

For the analysis of microcirculatory videos, we refrained from using a software-based analysis. This was due to the fact that most of the available software is calibrated for images retrieved from sublingual mucosa.

We analyzed only vessels with a diameter below 21 μm. They are considered to be capillaries and, therefore, most relevant for microcirculation and the evaluation of sepsis severity.

All microcirculatory recordings and evaluation sessions were performed by one and the same investigator in an offline session.

## Case presentation

The patient, a 54-year-old woman with a body mass index of 32.4, was admitted to our critical care unit because of respiratory insufficiency. Pre-existing conditions included stage IV arterial occlusive disease, recurrent panic attacks, and long-standing alcohol and nicotine abuse. The patient had a markedly reduced general condition with dehydration, cough without fever and sputum for about 2 weeks, and dry gangrene of the right foot.

**FIGURE 1:**
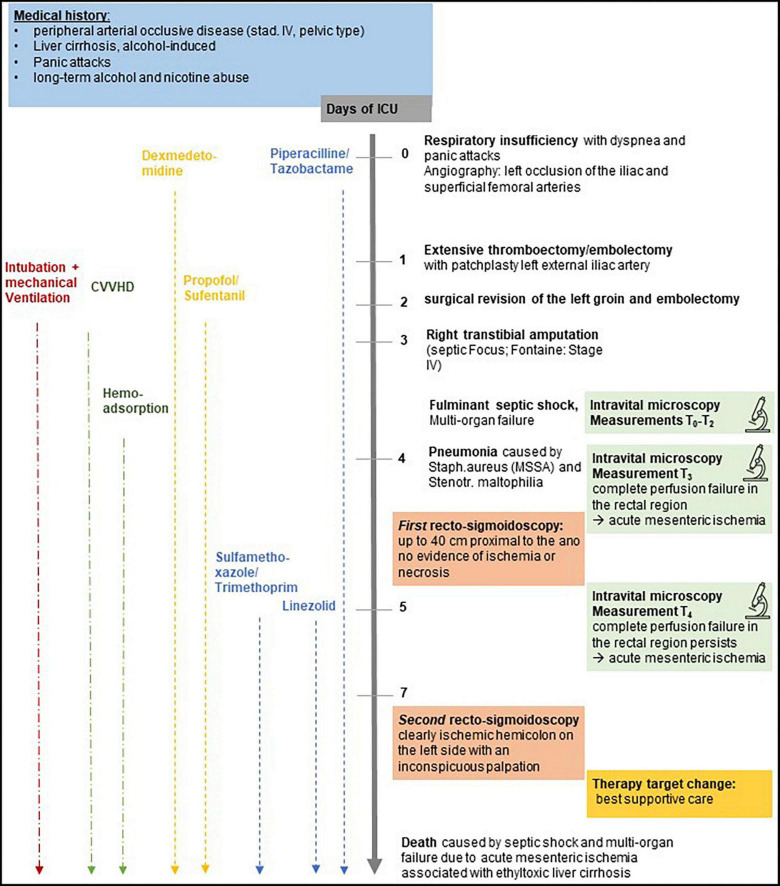
Timeline with relevant anamnestic, therapeutic, and operative data.

Computed Tomography (CT) angiography showed complete occlusion of the iliac and superficial femoral arteries on the left side and multiple stenoses of the common and internal iliac arteries on the right side. A timeline with relevant anamnestic, therapeutic, and operative data is presented in Figure 1.

After preoperative management with adjustment of volume and electrolyte balance on the first day in the intensive care unit (ICU), vascular surgery was performed for extensive thrombectomy/embolectomy with patch plasty of the left external iliac artery and debridement of the left groin.

Postoperatively, the patient was intubated and mechanical ventilation was continued under sedation with propofol, sufentanil, and dexmedetomidine in the ICU until the end of treatment. Hyperactive delirium with productive symptoms due to a known alcohol dependence required early pharmacological treatment. The other pre-existing conditions were treated according to the general therapeutic algorithm, such as therapeutic heparinization.

Sustained stabilization of the overall situation was not achieved. With the suspicion of sepsis, we started a comprehensive focus search and simultaneously initiated general sepsis therapy. On the third day in the hospital, we diagnosed septic shock (T_0_) and adjunctive hemoadsorption with CytoSorb^®^ was added to the ongoing renal replacement therapy (continuous venovenous hemodialysis [CVVHD]) that was started 24 h earlier. In addition to radiographic suspicion of pulmonary infiltration, the septic focus seemed to be the gangrenous area of the right foot. Since previous attempts of revascularization had failed, we performed a right transtibial amputation on the same day.

The subsequent clinical course (Table 1) was characterized by progressive septic multi-organ failure (renal, circulatory, coagulation, and hepatic) despite putative focal rehabilitation. The patient was included in the ongoing prospective study on microcirculation. The patients calculated Sequential Organ Failure Assessment (SOFA) score was 12 and the Acute physiology and chronic Health Evaluation (APACHE) II score was 32 with a predicted mortality of 86.66%. IVM of the oral and anal mucosa was performed.

**TABLE 1:** Laboratory and hemodynamic parameters and vasopressor doses during septic shock and microcirculatory assessments.

Parameter	0 h (T_0_)	4 h (T_1_)	8 h (T_2_)	15 h	24 h (T_3_)	39 h	48 h (T_4_)
APACHE II	32						
SOFA score	12				14		14
Interleukin-6 (pg/ml)	211.5	nd	nd	432.2	488.4	1034.0	nd
CRP (mg/l)	124.00	nd	nd	104.4	102.2	108.8	nd
Procalcitonin (μg/l)	0.17	nd	nd	0.14	0.15	0.29	nd
Leukocytes (g/l)	10.50	nd	nd	8.4	11.5	13.6	19.2
Hemoglobin (g/l)	82	nd	nd	79	81	98	111
Hematocrit (l/l)	0.24	nd	nd	0.23	0.20	0.28	0.29
Thrombocytes (g/l)	138.00	nd	nd	41	40	17	19
ASAT (U/l)	87.00	nd	nd	65	nd	104	nd
ALAT (U/l)	31.00	nd	nd	22	nd	24	nd
Bilirubin (mg/dl)	0.20	nd	nd	0.15	nd	0.40	nd
Alkaline phosphatase (U/l)	119.00	nd	nd	112	nd	119	nd
Cholinesterase (U/l)	1249	nd	nd	1,266	nd	1275	nd
Gamma-GT (U/l)	41	nd	nd	47	nd	62	nd
LDH (U/l)	538	nd	nd	493	nd	416	nd
CPK	nd	nd	nd	nd	nd	nd	nd
Myoglobin (μg/l)	nd	nd	nd	510	nd	2813.6	nd
Albumin (g/l)	19.50	nd	nd	13.3	3.3	14.3	nd
INR	1.10	nd	nd	1.39	1.38	1.50	1.18
Creatinine (mg/dl)	0.31	nd	nd	0.25	nd	0.27	nd
Arterial pH	7.23	7.29	7.43	7.37	7.29	7.39	7.32
pCO2 (mmHg)	48.7	53.4	37.1	40.8	50	47	56
pO2 (mmHg)	78.4	43.9	156	139	74.7	69.8	57
HCO3 (mmol/l)	18.6	22.8	24.1	22.9	22.2	26.8	25.4
BE (mmol/l)	–7	–1.7	–0.5	–1.9	–2.6	2.6	1.2
cHb (g/dl)	7.8	7.8	7.7	7.9	6.5	9.8	9.2
Hkt	23	23	23	23	19	29	27
Central venous SO2 (%)	86.9	98.7	62.7	nd	53.1	93.8	61.4
Na^+^ (mmol/l)	136	137	137	136	137	137	137
K^+^ (mmol/l)	3.69	3.51	3.56	4.48	4.72	3.94	3.76
CA^++^ (mmol/l)	1.11	1.1	0.9	1.01	1.06	1.09	1.13
CL^–^ (mmol/l)	104	102	106	106	109	108	107
Anion gap (mmol/l)	15.1	13.8	10.7	11.5	9.2	4.6	5.4
Lactate (mmol/l)	8.23	6.4	5.23	6.36	6.84	2.32	1.93
Glucose (g/dl)	233	175	111	92	114	148	141
Norepinephrine (μg/kg/min)	0.392	0.314	0.471	0.667	0.706	0.157	0.175
Argipressin (IE/h)	0.8	0.8	0.8	0.8	0.96	0.96	0.96
MAP (mmHg)	79	80	83	73	74	88	101
Heart rate	106	92	97	97	101	83	110

CRP, C-reactive-protein; INR, international normalized ratio; ASAT, aspartate-aminotransferase; ALAT, alanine-aminotransferase; Gamma-GT, gamma-glutamyltransferase; LDH, lactate dehydrogenase; CPK, creatinine phosphokinase; pCO2, partial pressure of carbon dioxide; pO2, partial pressure of oxygen; HCO3, bicarbonate; BE, base excess; MAP, mean arterial pressure; cHb, hemoglobin; Hkt, hematocrit; SO2: oxygen saturation; nd, no data. Laboratory values after 15 h and 39 h represent routine parameters determined at the standard ICU time point.

**FIGURE 2:**
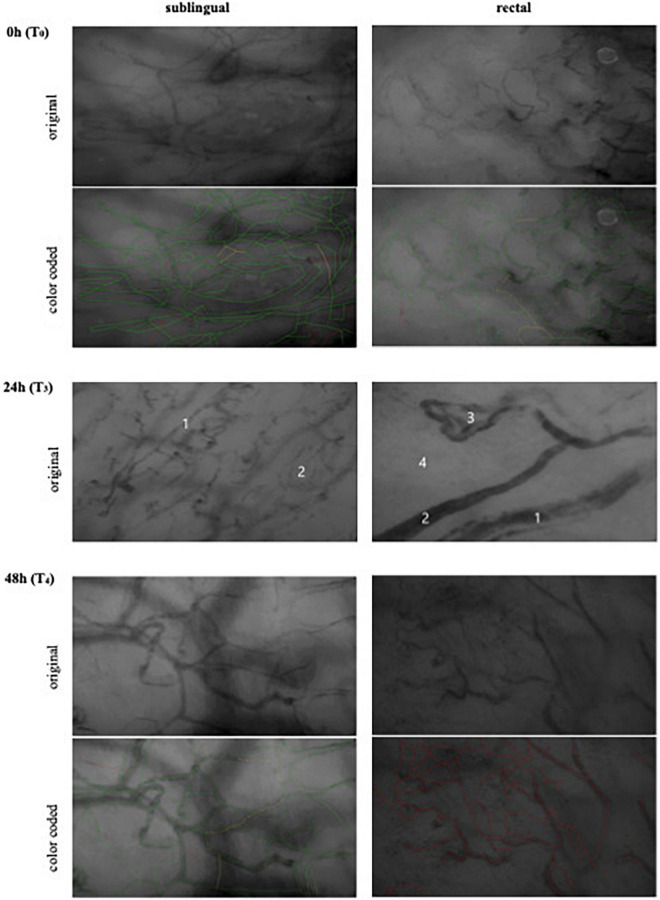
Intravital microscopy measurements. Zero hour (T_0_), day 3 after ICU admission. Onset of septic shock with generally compromised but the microcirculation was preserved. Twenty four hours (T_3_), day 4 after ICU admission. Sublingual 24 h (T_3_): the overall vessel density is higher than in the rectal measurement. Sublingual description: (1): Capillary of about 10 μm diameter. Due to hemodilution caused by the fluid resuscitation, there are heterogeneous aspects in the run of this and other capillaries. In contrast to the rectal vessels, these vessels showed flow after carefully releasing pressure which the probe applied to the tissue. Additionally, the diameter of this vessel only allows one, at most two erythrocytes to move through, so that heterogeneous absorption patterns and even discontinuities can be explained by the folding of erythrocytes. An unimpaired vessel of 70 μm on the other hand should have plenty of moving cells, so that discontinuity and irreversible stagnant flow should not arise. (2): Area with normal vessel density and visible capillaries. Rectal description: (1): Arteriole of about 50 μm diameter. The vessel appears to have multiple lumina as well as a heterogeneous absorption along its run. We suspect this to represent clotting. (2): Arteriole of about 20 μm diameter. The lumen of this vessel is brighter at the center than at the walls, which may also represent clotting. (3): Loop of an arteriole, approximately 20–30 μm. Heterogeneous absorption, apparently multiple lumina and even discontinued run. (4): Area without visible capillaries. All vessels presented no flow. Even with the lowest possible pressure that would still allow seeing the vessels, it was not possible to restore flow, which makes a pressure artifact highly unlikely. Forty eight hours (T_4_), day 5 after ICU admission. Complete rectal perfusion failure. Color code (based on the MFI): Green: constant flow; Yellow: intermittent/sluggish; Red: no flow.

At T_0_–T_2_, the measured perfusion of the sublingual and rectal mucosa was consistent with a general impairment of the microcirculation due to septic shock. Approximately 24 h after the onset of septic shock (T_3_), perfusion of the oral mucosa was still good, but in the rectal region, an almost complete perfusion failure was observed, suggesting an AMI (Figure 2). The corresponding intravital microscopy parameters and lactate values are shown in Table 2.

**TABLE 2:** Intravital microscopy parameters and lactate values 0, 4, 8, 24, and 48 h after onset of septic shock.

Hour after onset of septic shock	De-Backer score (n/mm)	PPV (%)	PVD (mm/mm^2^)	MFI	Vessel crossings	Perfused vessel crossings	Lactate (mmol/l)
**Measurement location: sublingual**							
0 (T_0_)	10.1	0.89	8.99	2.85	51.2	45.6	8.23
4 (T_1_)	6.82	0.87	5.96	2.8	34.6	30.2	6.4
8 (T_2)_	9.01	0.96	8.61	3	45.67	43.67	5.23
24 (T_3_)	9.92	0.95	9.47	2.93	50.29	48	6.84
48 (T_4_)	6.77	0.95	6.41	2.79	34.33	32.5	1.93
**Measurement location: rectal**							
0 (T_0_)	10.16	0.87	8.84	2.63	51.5	44.83	8.23
4 (T_1_)	12.11	0.97	11.72	3	61.4	59.4	6.4
8 (T_2)_	14.24	0.70	9.94	2	72.2	50.4	5.23
24 (T_3_)	Almost complete perfusion failure in the rectal						6.84
48 (T_4_)	region consistent with acute mesenteric ischemia						1.93

PPV, percentage of perfused vessels; PVD, perfused vessel density; MFI, microvascular flow index.

**FIGURE 3:**
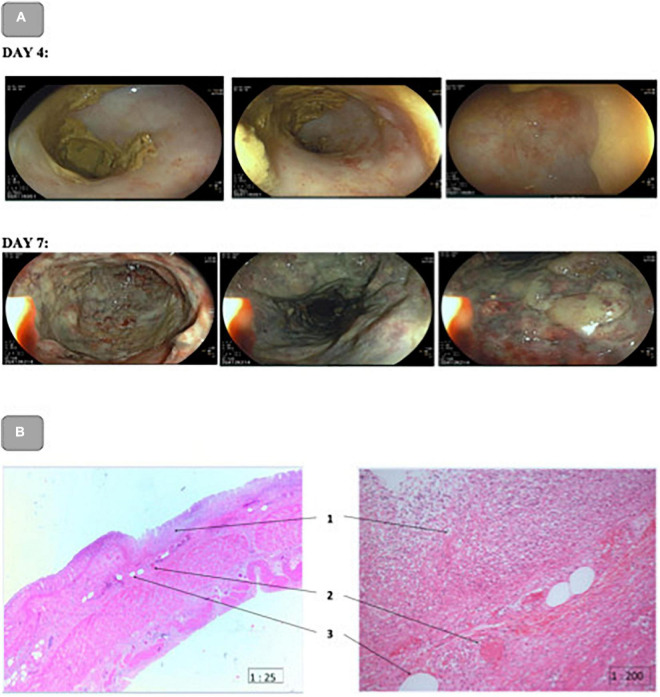
**(A)**: Rectosigmoidoscopy on day 4 and Colonoscopy on day 7 after ICU admission. Day 4: No signs of ischemia or necrosis up to 40 cm after ano. The bowel wall appeared vital. Day 7: Markedly ischemic hemic colon on the left. Rectum appeared to be conditionally vital, the colon from 20 cm to approximately 70 cm showed extensive mucosal necrosis with presumed involvement of all wall layers. **(B)**: Pathohistological picture of the upper rectum. H&E staining magnification 25× (left) 200× (right). (1): Extensive mucosal and partial rectal wall necrosis with granulocytic demarcation; (2): Eosinophilic intravascular fibrin thrombus; (3): Fatty vacuoles.

The subsequent rectosigmoidoscopy showed no signs of ischemia or necrosis up to 40 cm after ano. The bowel wall appeared vital (Figure 3A).

During the next 24 h (T_3_–T_4_), there was a discrete tendency toward clinical stabilization. The serum lactate level decreased from 6.8 mmol/l to <2 mmol/l. Possible previous episodes of mesenteric ischemia, such as diarrhea, weight loss, and postprandial pain, were not known anamnestically. From the visceral surgery point of view, there was no indication for additional diagnostics, such as angiography, CT angiography, and laparotomy.

In contrast, the IVM showed a similar picture of complete rectal perfusion failure also after 48 h (T_4_) after onset of septic shock (Day 5) indicating persisting AMI (Figure 2).

The second, control recto-sigmoidoscopy on day 7 revealed a markedly ischemic hemic colon on the left side with unremarkable abdominal palpation. The rectum appeared to be conditionally vital, the colon from 20 cm to approximately 70 cm showed extensive mucosal necrosis with presumed involvement of all wall layers (Figure 3A). Therefore, an emergency indication for laparotomy was made, which required critical interdisciplinary decision-making.

The situation was discussed in detail with the family. The need for bowel resection with the creation of a terminal colostomy/ileostomy was explained.

After thorough consideration of all arguments and with special regard to the patient’s presumed will, it was concluded that the patient would not have given her consent to this operation. The therapy was limited to supportive care. On the late evening of day 7 after admission to the ICU, 5 days after onset of septic shock, the patient died from septic shock with multi-organ failure due to AMI associated with ethyl toxic liver cirrhosis. The pathological anatomical diagnosis was gangrenous sigmoiditis with purulent peritonitis and ischemic colopathy due to high-grade outflow stenosis (2 mm) of the inferior mesenteric artery (Figure 3B) consistent with an acute on chronic mesenteric ischemia.

## Discussion

The initial clinical impression that complicated gangrene of the right lower leg had caused the septic shock was deceptive. Rather, it must be assumed that the perfusion deficit of the superior mesenteric artery and the complete occlusion of the inferior mesenteric artery caused the sepsis. It could not be compensated by the residual flow in the area of the middle and inferior rectal artery. The resulting complete intestinal wall necrosis led to the persistence of septic shock with the need for high-dose vasopressor support that further aggravated the situation. The pathohistological findings indicate total perfusion failure in this area for approximately 2–3 days (Figure 3B). This course is clearly confirmed by the IVM findings. Initially, despite the multiple organ replacement (kidney, lung, and liver) required due to multi organ failure, the microcirculation was relatively well-preserved. As early as 24 h after the onset of septic shock, we saw marked differences in perfusion indices. While the sublingual mucosa showed a still sufficient microcirculation, the rectal perfusion was no longer visible microscopically (no flow).

In patients with abdominal sepsis, dissociation between sublingual and intestinal perfusion has been reported (26). This is not evident in the case described here. Rather, MFI and PPV did not differ between sublingual and rectal measurement sites within the first 8 h, i.e., a clear difference between the measurement sites in terms of blood flow or percentage of perfused vessels was not detectable. Several aspects may explain this. First, the comparability with the study of Edul et al. (26) is limited. The patient described here had significantly more severe disease, as indicated by the APACHE II score of 32 and the SOFA score of 12–14 during the course [compared with Edul et al. (26) APACHE II: 20; SOFA:7]. While norepinephrine doses were comparable, we had to use additional vasopressin. In conclusion, the “better” microcirculation in our case during the course could be due to immunomodulation by adjuvant hemoadsorption. This all has to be seen in the context of the impaired liver function in the multi organ dysfunction syndrome (see Table 2) and could be another explanation for the apparent discrepancy between the adequate blood flow measured sublingually and rectally (within the first 8 h) and the high serum lactate concentration. After 24 h, no blood flow was detected rectally, even in larger vessels (>21 μm).

In contrast, the established clinical and endoscopic examinations performed did not reveal a clinically significant reduction of perfusion. This once again highlights the complexity of detecting and managing an AMI, as the clinical approach requires rapid diagnosis and surgical therapy (13).

Especially acute or chronic mesenteric ischemia, as seen in this patient, remains one of the major challenges in abdominal surgery. Visible light spectroscopy is one of the strongest predictors of the restoration of vascular patency in patients suffering from chronic gastrointestinal ischemia (27). In this case, no sign of inferior mesenteric artery stenosis was described in initial CT angiography whereas the autopsy revealed a high-grade outflow stenosis. A proven stenosis of the left iliac arteries might have led to the assumption of impaired perfusion in the flow area of the middle or inferior rectal artery. With the persistence of septic shock and due to results of IVM, recto-sigmoidoscopy was performed. This endoscopy in the absence of peritoneal signs is easily feasible in ICU and a standard in the diagnosis of ischemic colitis (28). Endoscopic findings, that strongly implicate ischemic colitis, are, for example, edematous and fragile mucosa, longitudinal ulceration, segmental erythema, petechial hemorrhage, or scattered erosions (29, 30). Still, colonoscopy may have a time delay in the clinical presentation of ischemic mucosa (31) of about 4 h (16). In this case, no signs of ischemic colitis were seen, possibly because typical mucosal changes had not yet formed. Retrospectively, a second CT-angiography might have revealed a severe stenosis of the inferior mesenteric artery caused by an acute on chronic mesenteric ischemia.

Nonetheless, conservative therapy such as hemodynamic stabilization appeared to be sufficient. There were no clinical findings that suggested acute abdomen. The serum lactate concentration even decreased to 1.34 mmol/l 50 h after the onset of septic shock. Probably this was related to a completely occluded arterial inflow and the resulting lack of venous reflow with a relevant concentration of lactate. Furthermore, the fast decrease of lactate levels can be explained by progressive necrosis and complete breakdown of the intermediate metabolism.

Managing acute on chronic mesenteric ischemia is a multi-modular task, especially in sedated and ventilated intensive care patients with a high risk of low mesenteric blood flow due to the necessity of hemodynamic support.

Paraclinically, the serum lactate concentration is the recommended and most commonly used parameter (13, 32). However, it is affected by a variety of factors like liver function, hemodynamic situation, and laboratory chemical specification and is, therefore, very non-specific for the diagnosis of mesenteric ischemia (16, 32, 33).

Special vascular diagnostic methods utilize duplex ultrasound examination (DUS) or angiographic procedures. DUS can be performed bedside and be helpful in the emergency department if the clinic is appropriate (34). CT angiography is the main recommended investigation for ischemic bowel disease. It allows assessment of the abdominal vessels (arteries and veins), bowel wall thickness, bowel loops, and other intra-abdominal organs, among others (33, 35). Limiting factors include the need for in-clinic transport and the application of a contrast medium in critically ill patients.

Because of the significant differences between the vessel architecture of sublingual mucosa and the rectal mucosa comparability of the sublingual and the rectal De Backer scores is limited. However, a comparison of De Backer scores obtained at the same location but at different time points could be a feasible option.

AMI is a timecritical and life-threatening condition. Particularly in sedated and ventilated patients, the clinical diagnosis of AMI is complicated (15).

All methods used in this case have their own limitations. The clinic is as non-specific as various laboratory chemistry parameters. Angiographic diagnosis requires the provision of a highly specialized radiological infrastructure in addition to urgent, high-risk in-hospital transport (36). Endoscopy, which can be performed bedside, may not reveal pathological changes in the initial phase and carries the risk of iatrogenic perforation (37, 38) and tension pneumoperitoneum (39, 40).

From our view, IVM could have potentially accomplished the rapid diagnosis required in this case. IVM is not yet widely used probably because it is a complex procedure with multiple possibilities of mismeasurement and misinterpretation (11).

Otherwise, it has distinct advantages in terms of accurate assessment of local perfusion conditions. IVM can be performed bedside and repeatedly with little effort and, in the hands of the skilled, can provide an early indication of mesenteric ischemia. However, sublingual assessment alone would not have been sufficient in this case because the failure of perfusion in the inferior rectal artery would not have been detected earlier otherwise had the obstruction been localized in other, more oral parts of the intestine, a rectal measurement might also have been insufficient.

In addition, a trend toward dissociation between the sublingual and intestinal microcirculation is known (26), implying that the procedure we describe with sublingual and rectal IVM may be a useful clinical approach for a general assessment of microcirculation compared with intestinal conditions.

In fact, IVM is not mentioned in the current guidelines for AMI (13) and we suggest that the value of IVM should be re-evaluated in this context. In our perspective, it should be part of a diagnostic approach to allow early surgical treatment and to improve the poor prognosis of AMI, especially in critically ill ICU patients. It might also be worth considering if IVM can help to evaluate the circulation in an anastomosis right in the operation room.

## Data availability statement

The original contributions presented in this study are included in the article/supplementary material, further inquiries can be directed to the corresponding author.

## Ethics statement

The study involving human participants was registered in the German Clinical Trials Register (DRKS00017211) and was reviewed and approved by the Institutional Ethics Committee of the Ruhr-University Bochum (reference number: 2019-440_2). Written informed consent was obtained from the individuals’ legal guardian/next of kin, for the publication of any potentially identifiable images or data included in this article.

## Author contributions

JP was directly involved in the study inclusion and performance of measurements and was mainly responsible for writing the manuscript. CK analyzed and interpreted the data regarding surgical aspects and reworked the manuscript. ES performed literature research, analyzed the case, and reviewed the manuscript. SA performed IVM measurements at all time points and participated in the preparation of the manuscript. AW performed the pathological and histological examinations and revised the manuscript from a pathological point of view. DH analyzed and interpreted the data from an anesthesiological point of view and reworked the manuscript. TK treated the patient in ICU, analyzed and interpreted the data, and was a major contributor in writing the manuscript. All authors have read and approved the manuscript.

## Abbreviations

AMI: acute mesenteric ischemia
DUS: duplex ultrasound examination
ICU: intensive care unit
IVM: intravital microscopy
MFI: microvascular flow index
SDF: sidestream dark field imaging.
